# Differentially expressed long non-coding RNAs and mRNAs of cadmium exposure on learning disability of offspring rats

**DOI:** 10.1186/s40001-024-01663-4

**Published:** 2024-01-29

**Authors:** Hui Liu, Xichen Wan, Liyun Yao, Qihan Zhao, Yong Yang, Hongtao Liu, Jun Shang, Fanfan Zeng, Xin Wang, Shaoxin Huang

**Affiliations:** 1 School of Nursing, Jiujiang University, Jiujiang, 332000 China; 2 School of Medicine, Jiujiang University, Jiujiang, 332000 China; 3 SpecAlly Life Technology Co., Ltd., Wuhan, 430070 China; 4 Wuhan Institute of Biotechnology, Wuhan, 430070 China; 5 Department of Neurosurgery, The First Affiliated Hospital of Nanchang University, Nanchang, 330000 China

**Keywords:** Cadmium, Cognitive ability, Molecular mechanism, Gene chip, Offspring

## Abstract

**Background:**

Cadmium (Cd) exposure has been found to have detrimental effects on the development of the central nervous system and cognitive ability in children. However, there is ongoing debate regarding the impact of maternal Cd exposure on the cognitive ability of offspring. In this study, we aimed to investigate the mechanisms underlying the influence of maternal Cd exposure on the cognitive ability of offspring rats.

**Methods:**

Here, we constructed a model of cadmium poisoning in first-generation rats through gavage. The cognitive and memory abilities of its offspring were evaluated by water maze experiment. Then, we used the gene chip to find out the key genes, and we performed qRT-PCR detection of these genes. Subsequently, enrichment analysis was employed to identify pathways. Finally, we constructed a co-expression network consisting of LncRNAs and mRNAs to elucidate the biological functions and regulatory mechanisms of LncRNAs.

**Results:**

The results of the water maze trial demonstrated that the offspring of rats exposed to cadmium in the first generation had reduced cognitive and memory abilities. Through an analysis of gene expression in the hippocampus of the cadmium-treated rats' offspring and the control group, we identified a correlation between the islet secretion pathway and the cognitive impairment observed in the offspring. Utilizing various algorithms, we identified Cpa1 and Prss1 as potential key genes associated with the cognitive impairment caused by cadmium. The results of qRT-PCR demonstrated a decrease in the expression levels of these genes in the hippocampus of the cadmium-treated rats’ offspring. In addition, in the co-expression network, we observed that Cpa1 was co-expressed with 11 LncRNAs, while Prss1 was associated with 4 unexplored LncRNAs. Furthermore, we conducted an analysis to examine the relationship between Cpa1, Prss1-related transcription factors, and LncRNAs.

**Conclusion:**

Overall, this study provides novel insights into the molecular effects of first generation Cd exposure on the cognitive ability of offspring. The target genes and signaling pathways investigated in this study could serve as potential targets for improving neurodevelopment and cognitive ability in children.

**Supplementary Information:**

The online version contains supplementary material available at 10.1186/s40001-024-01663-4.

## Introduction

Cadmium (Cd) is not just a recognized carcinogen; it is also classified as a potential agent with toxic effects on the development of the nervous system [1]. Cd can have multiple adverse effects on the central nervous system, often presenting as memory loss and psychiatric disorders [2]. Infants and children are particularly vulnerable to the toxic effects of cadmium [3, 4]. It is possible for cadmium to cross the placental barrier from the mother to the fetus [5]. Based on the study by Chow et al., they hypothesized that exposure to cadmium during prenatal development could hinder the differentiation of neurons and the formation of axons in the embryonic brain of zebrafish [6]. High levels of Cd accumulated in the mother's body could potentially cause harm to both the mother and the newborn child [1]. Numerous research studies have demonstrated that cadmium directly impacts the development of the central nervous system and is linked to behavioral and cognitive dysfunction, including early impairments in learning and memory among children [7]. Cohort studies conducted in various regions have discovered a negative association between prenatal exposure to cadmium and intelligence scores in children [8, 9]. However, evidence concerning the relationship between maternal Cd exposure and neurological development from population studies remains limited [10]. Several relevant studies have summarized conflicting conclusions, suggesting that the effects of perinatal cadmium exposure on cognitive abilities in offspring were not significant [11]. The association between prenatal exposure to Cd and neurodevelopment is still unclear, necessitating further investigation.

The neural development of second generation remains poorly understood in relation to the effect of first generation cadmium exposure, according to research [3]. Currently, the neurotoxicity of cadmium on second generation is believed to be attributable to epigenetic or oxidative stress, as well as the blood–brain barrier [12, 13]. Yu Mei Zhang has argued that Cd has a detrimental impact on mitochondrial swelling, crista rupture and loss, nissl body dissolution, degeneration of organelles and vacuoles, disappearance of cell membrane, concentration of nuclear chromatin, and modification of lipid peroxidation and ultrastructure in rat brain due to perinatal exposure to low doses of Cd [14]. In addition, some studies indicate that Cd may alter fetal development through an epigenetic mechanism, resulting in changes in gene expression during fetal development [15].

In order to investigate the cognitive ability of second generation rats (F1 rats) exposed to cadmium through their first generation (F0 rats), we developed maternal cadmium contact models and evaluated the cognitive ability of the second generation rats using a water maze experiment. The water maze (WM) is a widely used paradigm and a highly effective method for assessing chronic cognitive impairment in animals [16]. Through an analysis of the hippocampal tissue from cadmium-exposed rats and control rats in the second generation, employing a chip microarray, we sought to uncover the potential molecular mechanisms contributing to cognitive impairment caused by exposure to first generation cadmium.

## Methods

### Laboratory animals and study design

All animal experiments were conducted according to accepted animal welfare standards governed by the experimental Animal Welfare Committee of Jiujiang University. The rats were intragastrically administered normal saline (control group; *n* = 12; 6 males and 6 females) or 6 mg/kg of CdCl_2_ (Cd group; *n* = 12; 6 males and 6 females) for 8 weeks; Cadmium exposure dose was chosen according to the constructed dose of the animal model presented in the previous paper [17]. Cadmium chloride (CdCl_2_) was obtained from Shanghai McLean Biochemical Co., Ltd. (Shanghai, China). To maintain a consistent volume of the intragastric solution, each intragastric administration involved a fixed volume of 1 ml. Prior to gavage, the rats were weighed, and then the CdCl_2_ gavage solution was prepared with the appropriate concentration based on their body weight, and the CdCl2 solution was diluted with saline. The F1 rats drank ultra-pure water and were exposed to a 12-h light/dark cycle. The temperature was maintained at 20–25 °C and humidity at 50–60%. The feeding equipment and environment were strictly controlled to prevent Cd contamination. After 8 weeks of intragastric administration of saline or CdCl_2,_ the rats in each group were allowed to mate. Female rats that were determined to have mated were separated and given normal food and drinking water. Following natural delivery, when the body weight of the F1 rats reached roughly 100 g, the F0 rats were sacrificed for additional experiments, such as atomic absorption spectroscopy to determine Cd levels.

**Fig. 1:**
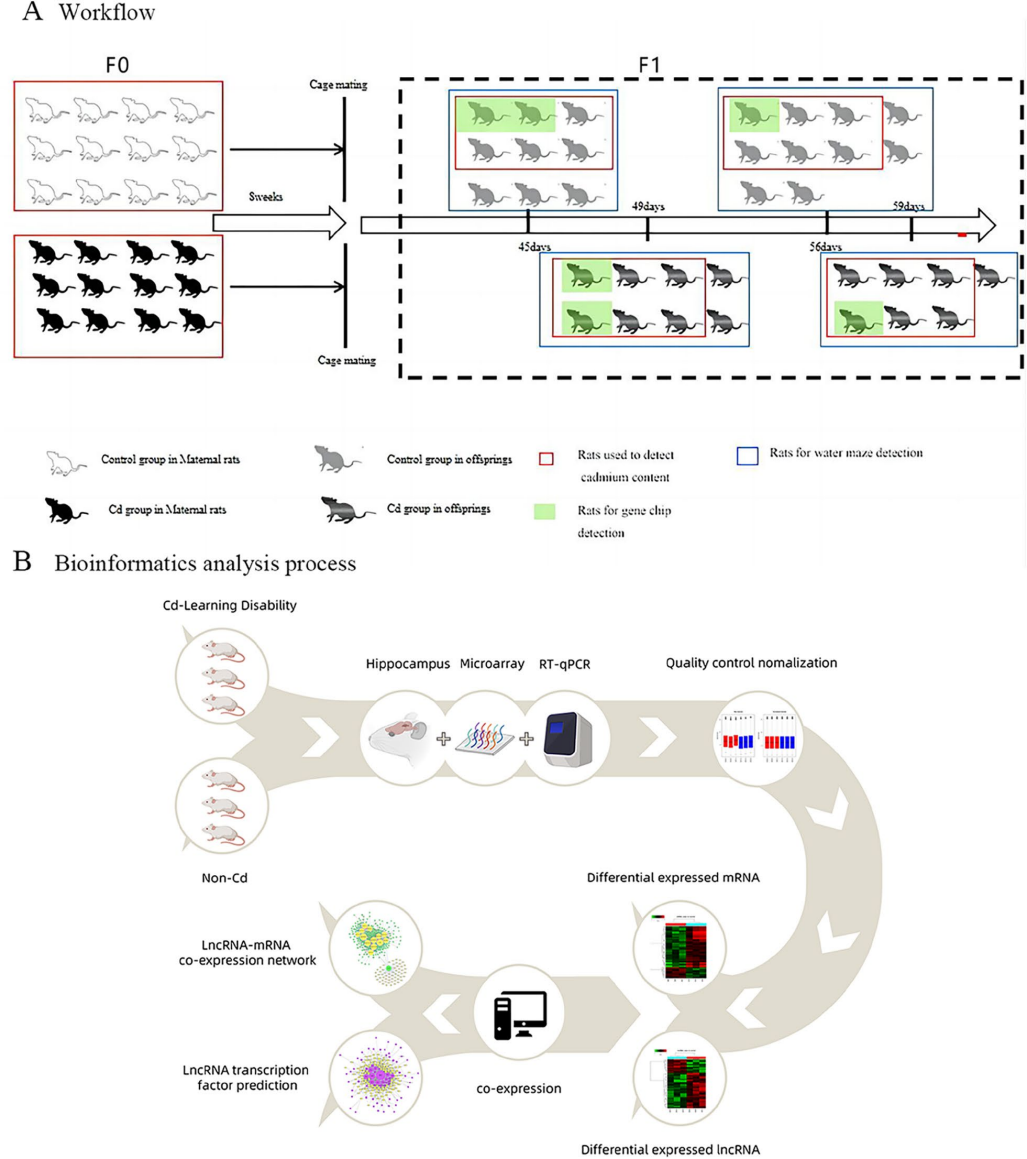
Overview of the study design. **A** Workflow; **B** Bioinformatics analysis process

The specific experimental design was shown in the Fig. 1. The F1 rats were used for cadmium detection, water maze experiment and microarray analysis of mRNA and LncRNA.

### Morris water maze experiment

19 F1 rats (10 males and 9 females) from the control group and 15 F1 rats (6 males and 9 females) from the Cd group underwent the Morris Water Maze test (SLY-WMS; Beijing Shunyu Musical Instrument Co., Ltd., Beijing, China) to evaluate spatial learning and memory abilities. The labyrinth consisted of an 8-cm-diameter platform and 90-cm-diameter pool filled with opaque water (22–25 °C), with a depth of 40 cm. The pool was divided into four quadrants.

#### Positioning navigation experiment

The experiment lasted for 6 days. On the first day, before the positioning navigation experiment test, the F1 rats were put on the platform to adapt for 15 s, and there was no adaptation process before the subsequent tests. After the test, no matter whether the F1 rats can successfully find the platform, put the F1 rats on the platform and let them stay for 10 s (adaptation). At the beginning of the experiment, the platform was installed 1 cm below the SE quadrant water surface. Each animal is tested 4 times a day. The first and fourth tests introduced water from the opposite quadrant (NW) of the target quadrant (SE). The time from water inflow to successful platform search is recorded as the escape latency. The experiment was stopped when the F1 rats found and boarded the platform or the experiment lasted more than 60 s. Let the F1 rats rest for 1 min and then conduct the next experiment. The escape latency time, average swimming speed (average speed) and total swimming distance were recorded.

#### Space search experiment

It will be carried out on the seventh day after the end of the six day positioning voyage. After the platform is removed from Morris water maze equipment, the F1 rats in the NW quadrant will be placed in water, and the number of times the F1 rats cross the original position of the platform, the average swimming speed of the F1 rats, the time ratio of the F1 rats in the original platform quadrant and the travel ratio (i.e., the target percentage), the time percentage of the quadrant and the target distance of the F1 rats in the original platform quadrant will be observed and recorded within 60 s The total distance of swimming and the spatial memory ability of evaluation.

#### Working memory experiment

The platforms in Morris water maze are installed in the NE, NW, SW and SE quadrants successively every day. Each F1 rat was tested 4 times a day. For the first and fourth tests, the rats enter from the opposite quadrant of the target quadrant. For the second and third tests, the entering quadrant is selected by random method (excluding the target quadrant and the opposite quadrant). The F1 rats were not acclimatized before each experiment, and were acclimatized for 10 s after each experiment. Other test procedures were consistent with the positioning voyage test. The incubation period, average speed and total swimming distance were observed and recorded.

### RNA extraction and microarray

The total RNA, including small RNA, was extracted using TRIzol reagent (Invitrogen, Carlsbad, CA, USA), according to the manufacturer's instructions. Fluorescent dye (Cy3-dCTP)-labeled cDNA was prepared using the Eberwine linear RNA amplification method and subsequent enzymatic reactions. The CapitalBio Technology rat LncRNA array v1 is designed to have four identical arrays (8 × 60 K format) for each glass slide, each array containing about 30,254 rat mRNA probes and 22,088 LncRNA probes. The hybrid images were analyzed and the data were extracted by Agilent Feature Extraction v10.7 (Agilent Technologies, Inc.), and the data were then normalized and differentially analyzed using the Agilent GeneSpring software.

### The analysis of differential expression level of mRNA and lncRNA from microarray

The differential expression level of mRNA and lncRNA from microarray was analyzed by using a limma R package. After quantile normalization, raw signals from microarray were log2 transformed. Differential expression of an mRNA or lncRNA was defined by absolute value of fold change (FC) > 2 (|log2FC| > 1) and *P* value < 0.05 (Student’s t test). Kyoto Encyclopedia of Genes and Genomes (KEGG_https://www.genome.jp/kegg/) and Gene Ontology (GO_http://www.geneongoloty.org/) Analysis was performed to determine the function of differentially expressed mRNA in biological pathways and GO terminology (cut-off P<0.05). Subsequently, modules of interest were visualized using STRING software (string-db.org/cgi/input.pl). To identify the hub genes in networks, Cytoscape software (version 3.6.2; http://www.cytoscape.org/) was used to analyze the degree of connectivity and establish mRNA-hub gene networks. The cytoHubba application was used to identify the top 10 genes with the highest degree of connectivity according to the Maximal Clique Centrality (MCC), Closeness, and Degree algorithms. We screened the top 10 hub genes using each algorithm and created a Venn plot to identify significant hub genes; genes falling in the overlapping area of the Venn plot were considered common hub genes.

### LncRNA-mRNA co-expression analysis and transcription factor prediction

Co-expression analysis and transcription factor prediction co-expression analysis are used for correlation analysis of signal value trends of differentially expressed lncRNA and mRNA in all samples (experimental group and control group) after comparison. The screening criteria were correlation > 0.99 or < − 0.99, *P* < 0.05. The lncRNA/mRNA pairs that meet the above conditions are considered to have a co-expression relationship.

Transcription factor (TFs) prediction, using Match-1.0 Public (http://gene-regulation.com/pub/programs), which is a transcription factor prediction tool, used to predict TFs binding sites, 2000 bp upstream and 500 bp downstream. According to the co-expression results, each lncRNA starting site used Cytoscape to create a network diagram (http://www.cytoscape.org/download.php) software.

### Quantitative real-time PCR

Quantitative real-time PCR was performed to validate the dark red module results of the microarray experiments. The total RNA was extracted according to the manufacturer's protocol for each kit. RNA quality was evaluated using a NanoDrop 1000 spectrophotometer; an optical density of 260/280 ratio of approximately 1.8 indicated pure RNA. Reverse transcription was performed using the first strand cDNA synthesis kit according to the manufacturer's protocol. Real-time quantitative PCR was performed using ABI Power SYBR1 Green PCR Master Mix (Applied Biosystems, Foster City, CA, USA) to detect the relative expression of the first 10 up- and down-regulated mRNA positions. Relative mRNA expression levels were calculated using the 2-^ΔΔ^ Ct method and normalized to β-actin expression. The primers used are shown in Table 1.

**Table 1:** Primers for RT-qPCR

Primer name	Primer sequence (5ʹ–> 3ʹ)
Cpa1-F	ACCCCATGGTCAAGGGTAGA
Cpa1-R	ACATCAATGGGGATACCGGC
Amy1a-F	GGTGGTGAAGCAGTGTCAAG
Amy1a-R	TGACGCCATCGATGTTCACAG
Cpa2-F	CAACCAGCAAAGAGAGCGTG
Cpa2-R	ACTTCAGAGTTGGGCTTGGG
Prss1-F	AACCCAGACCTGCTCCAATG
Prss1-R	GCCAACACAAATCATGCTGCT
Cpb1-F	GGCCCAGGAGCTACAACAAT
Cpb1-R	AGAAGCCTGTATCCCGGAGT
Prss3-F	TCACTGTTACAAGACCCGCA
Prss3-R	TCGAGCATTGAGTTTCACAGGG
Pnlip-F	GACAAGAGCAACTTCGCACG
Pnlip-R	TGCCGTCATTTCGTTCCACT
Cela2a-F	GGCACCACACCTTCAATCCT
Cela2a-R	GGCTGGGAACAGATGGA ATG
Pnliprp1-F	ATCAACACTCGCTTCCTGCT
Pnliprp1-R	TCAACCACCCAGTTCTCCTC

### Determination of Cd in the hippocampus by atomic absorption

The F0 rats (*n* = 24) were divided into two groups of 12 (6 males and 6 females in both groups): normal saline (control group) and 6 mg/kg Cdcl_2_ (Cd group). When rats weighed about 100 g, normal saline or Cd was intragastrically administered for 8 weeks. Hippocampal tissue of the F0 rats was obtained to determine the Cd content. After the water maze experiment, F1 rats were sacrificed to determine the Cd content in hippocampal tissue. The removed hippocampal tissue was mixed with 500 μL of protein lysate and ground in a grinder until no obvious tissue structure was visible. The remaining 500 μL of lysate was transferred to a 2 ml polyethylene test tube, mixed with 500 μL of 65% nitric acid, and digested overnight at 65 °C. Cd content was quantified by graphite furnace atomic absorption spectrometry (AAnalyst 800; PerkinElmer, Inc., Shelton, CT, USA) and normalized to the total protein content.

## Results

### Cd exposure in F0 rats may impair learning and cognition in F1 rats

**Fig. 2:**
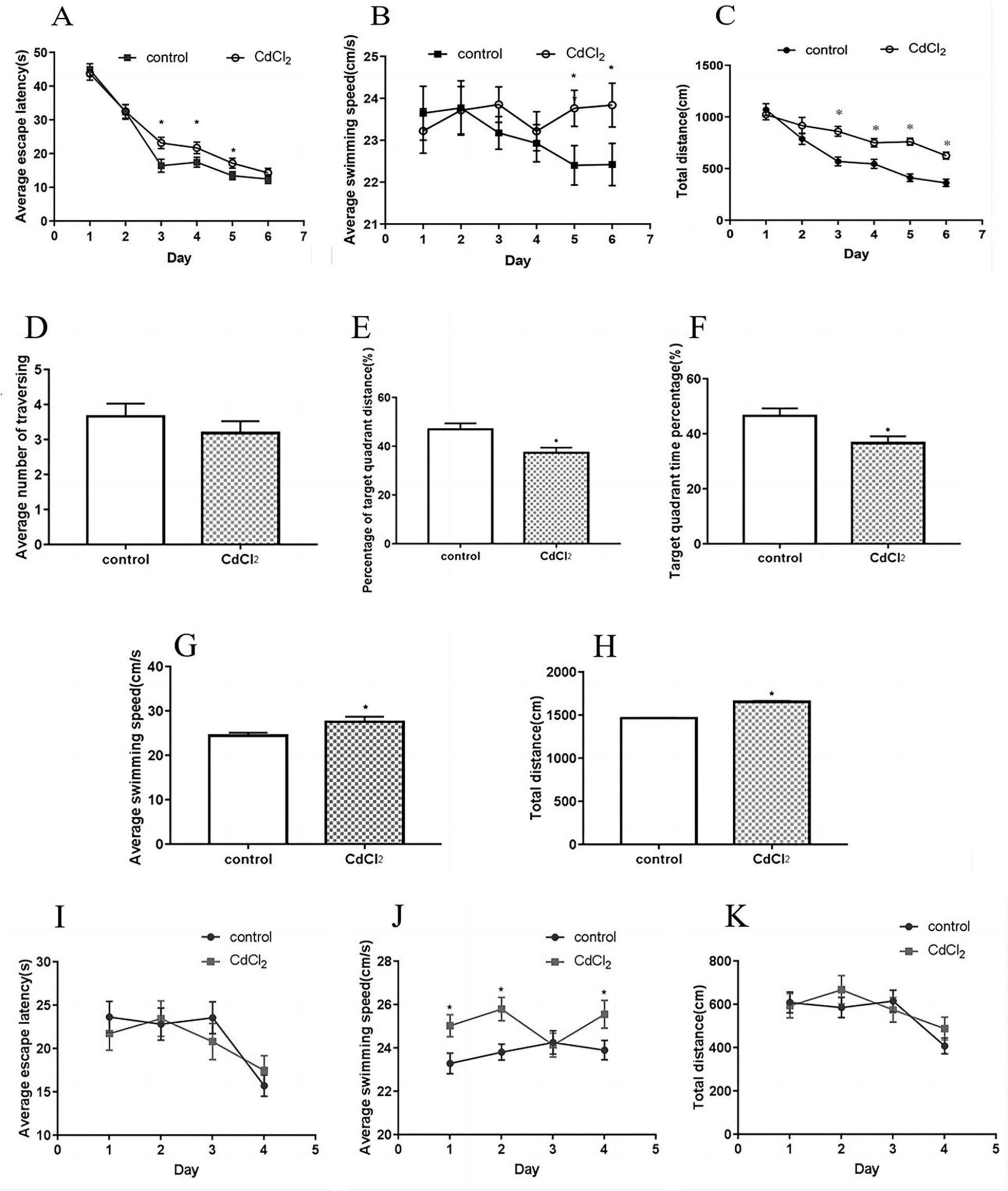
Effect of cadmium on cognition of rat offspring in water maze. Positioning cruise test: **A** Average escape latency; **B **Average swimming speed; **C** Total distance; Space Search Experimen: **D** Average number of traversing; **E** Percentage of target quadrant of distance; **F** Target quadrant time percentage; Working memory test: **I** Average escape latency; **J** Average swimming speed; **K** Total distance

In the positioning cruise test, the incubation period of both the experimental group and the control group decreased with an increase in training days (d1 ~ d6) (*P* ≤ 0.05). From the third day of training, the mean latency of F1 in the cadmium exposure group was significantly higher than that in the control group for three consecutive days (*P* ≤ 0.05, Fig. 2A). Interestingly, as the training days increased (d1 ~ d6), the daily average swimming speed of the control group decreased, while the experimental group showed an increasing trend. On the first day of the positioning navigation experiment, the average swimming speed of the F1 rats in the control group was significantly faster than that of the F1 rats in the experimental group (*P* ≤ 0.05, Fig. 2B). The average daily swimming speed of the F1 rats in the experimental group showed an increase starting from the second day of training. Notably, on the 5th and 6th training days, the swimming speed of the F1 rats in the experimental group exhibited a significant improvement as compared to the F1 rats in the control group (*P* ≤ 0.05, see Fig. 2C). In addition, there was observed heightened panic among the F1 rats when exposed to water. The average total distance covered by the F1 rats in the control group experienced a significant reduction starting from day 3 (*P* ≤ 0.05), and remained significantly lower than the corresponding swimming distance of the F1 rats in the experimental group for four consecutive days. Trend analysis revealed that the total swimming distance of the F1 rats in the experimental group surpassed that of the F1 rats in the control group (*P* ≤ 0.05).

Space Search Experiment: The results of the space search experiment showed that when the average number of F1 rats crossing the platform in the control group was taken as the control value, the correct number of F1 rats crossing the platform in the experimental group was slightly less than that in the control group (*P* ≤ 0.05, Fig. 2D). The percentage of target quadrant distance in the experimental group was significantly lower than that in the control group (*P* ≤ 0.05, Fig. 2E). The percentage of target quadrant time in the experimental group was also significantly lower than that in the control group (*P* ≤ 0.05, Fig. 2F). When the average swimming speed of the F1 rats of the control group was taken as the control value, the average swimming speed of the F1 rats of the experimental group was significantly higher than that of the control group (*P* ≤ 0.05, Fig. 2G). The total swimming distance of F1 rats in the experimental group was significantly higher than that in the control group (*P* ≤ 0.05, Fig. 2H).

Results of the working memory test: As the training days progressed (d1 ~ d4), the control group exhibited a consistent decline in the daily average escape latency of the F1 rats (*P* ≤ 0.05, Fig. 2I). Similarly, the experimental group also demonstrated a decreasing trend in average latency, albeit with slight fluctuations. Notably, on d1, d2, and d4, the experimental group exhibited a significantly higher average swimming speed compared to the control group (*P* ≤ 0.05, Fig. 2J). Moreover, the daily average total distances for both groups displayed a consistent decline (*P* ≤ 0.05, Fig. 2K).

### Sample analysis of differentially expressed lncRNAs and mRNAs from hippocampal tissues of F1 rat

The F0 rats were sacrificed after gavage of cadmium and the F1 rats would dead after completed the Morris water maze, and the content of cadmium in the hippocampus of F1 rats was determined by atomic absorption spectrometry. As Additional file 1: Figure S1A, comparison of cadmium levels in the brain tissue of F0 and F1 rats. Compared with the control group (normal saline group) administered with cadmium, there was no significant difference in the cadmium content in the hippocampal tissues of the F0 and F1 rats. This may be due to the influence of the blood–brain barrier. Although the cadmium failed to enter the hippocampus, it still produced functional damage. We used the CapitalBio Technology rat array v1 to analyze the differential gene expression in the hippocampal tissues of F1 rats, 3D PCA of LncRNAs (as shown in Additional file 1: Fig. S1B) and 3D PCA of mRNA (as shown in Additional file 1: Fig. S1C).

**Fig. 3:**
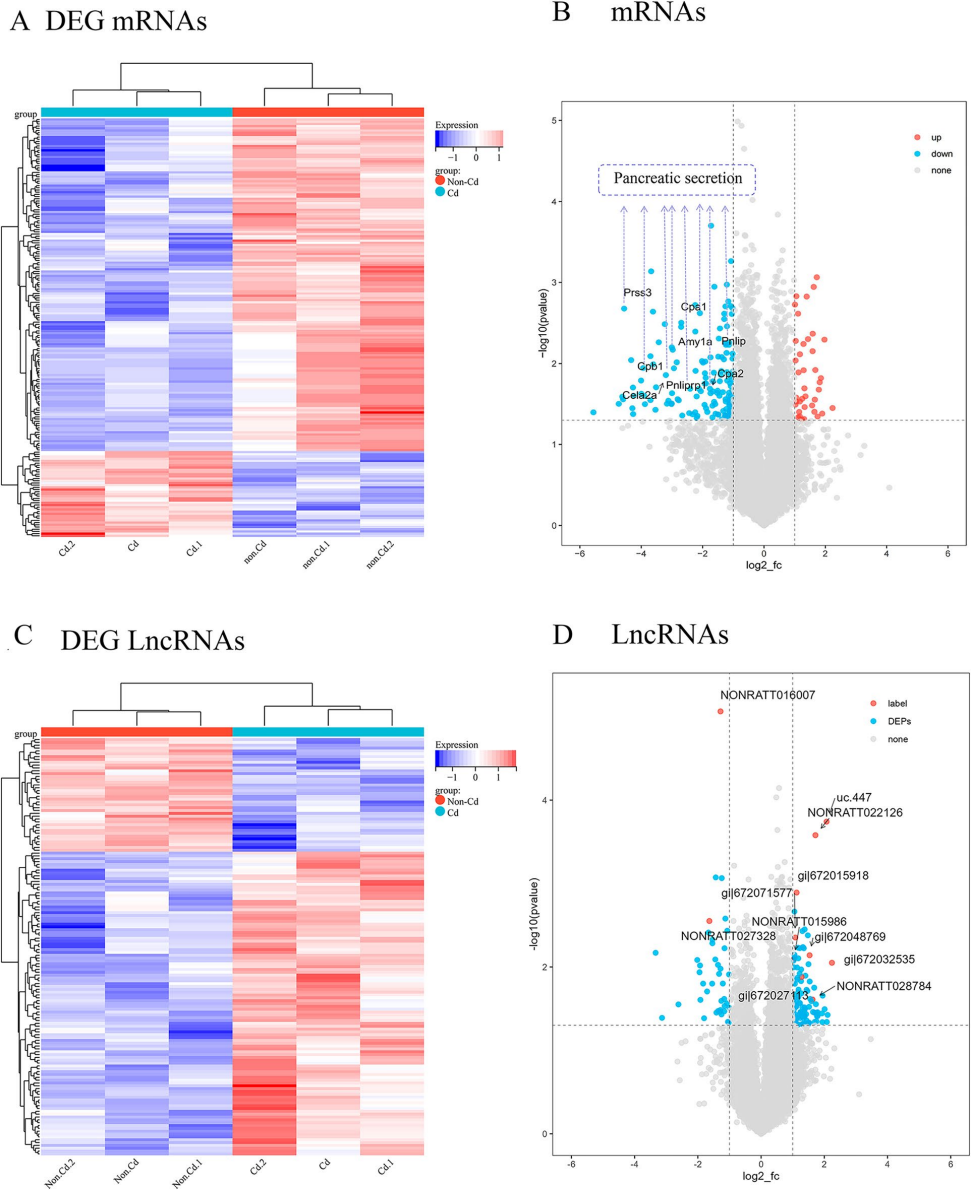
lncRNA and mRNA expression profiles. Heat map of differential expression (**A**) mRNAs and (**C**) LncRNAs. Each column represents a sample, while each row represents the degree of expression of a lncRNA or mRNA in a different sample. Red denotes high expression level, while blue denotes low expression.Volcano plots showing the up- and down-regulated **B** mRNAs and **D** lncRNA. The abscissa is -log10 (p value) and the ordinate is log2 (fold-change). Red denotes the up-regulated lncRNA/mRNAs, while blue denotes the down-regulated lncRNA/mRNAs, and black denotes no significant difference in gene expression. NC, non-treated control; lnc, long non-coding

As shown in Fig. 3, there were significant differences in the expression of mRNA and lncRNA in the hippocampus tissue of cadmium and control in F1 rats. Compared with the hippocampus of the F1 rats of the control group, the expression levels of 107 lncRNAs in the hippocampus of the F1 rats of the cadmium-exposed rats were up-regulated, while the expression levels of 40 lncRNAs were down-regulated. In addition, 40 mRNAs were up-regulated and 151 mRNAs were down-regulated. In Fig. 3A and C, the sample tree at the top represents similar clustering relationships between different samples. The color blocks at the top represent the expected grouping of samples manually set before the cluster analysis, and samples of the same color indicate that the experiment is expected to be a group. The p value obtained by the difference analysis and the FC value were combined to draw a Volcano plot to show the significant difference between the two groups of samples, mRNA (Fig. 3B)and LncRNA (Fig. 3D).

### GO analysis of LncRNA and mRNA in the hippocampus of F1 rats by maternal Cadmium exposure

**Fig. 4:**
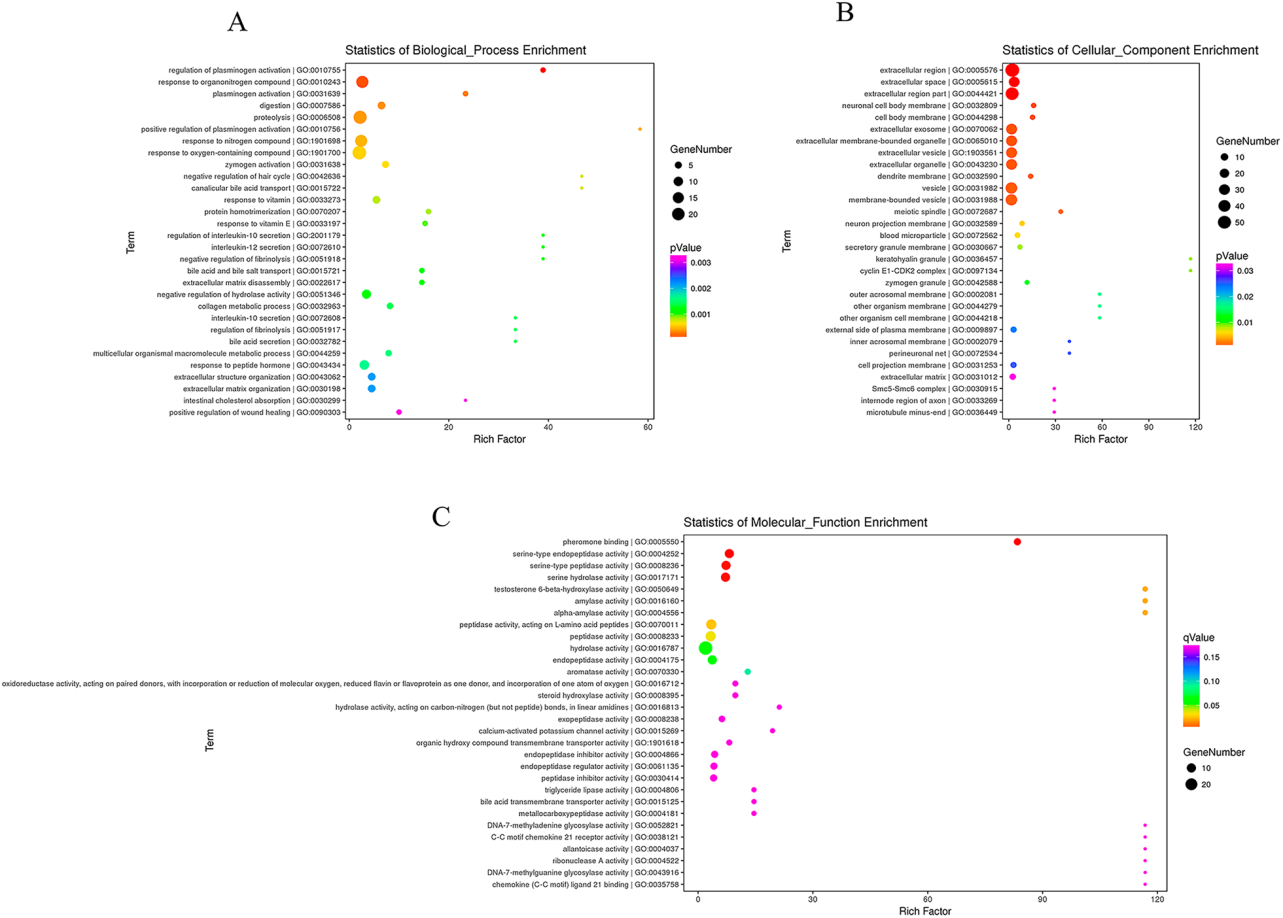
GO Analysis and Pathway Analysis of differentially Expressed mRNAs. Significantly enriched GO functions; the top 30 significantly enriched in **A** BP, **B** CC and **C** MF; the bubble map drawn according to the p value, the size of the bubble indicates the number of genes annotated to this function entry by differential genes, and the color Corresponding to the *P* value in the enrichment analysis results. The p value indicates the significance of GO enrichment in each group, and when *P* ≤ 0.05, it means that the differential gene is significantly enriched in the functional item

GO Enrichment analysis for targets to clarify the mechanism of maternal cadmium-exposed impairs learning cognitive ability in the second generation of offspring rats, and GO selected the top 30 significantly enriched functional entries from BP, MF, and CC respectively (Fig. 4A). Among the up-regulated mRNAs in the treatment group, 127 genes were related to “biological processes”, 26 were related to “cellular components”, and 55 were related to “molecular functions”. Among the down-regulated mRNAs, there were 225 groups of genes involved in “biological processes”, 25 genes involved in “cellular components”, and 14 genes involved in “molecular functions”. The results of bubble plot enrichment analysis showed that differential mRNAs were significantly enriched in biological pathways such as regulation of plasminogen activation, response to organic nitrogen compounds, plasminogen activator, digestion, and proteolysis (Fig. 4B). These differential genes were mainly concentrated in the extracellular, neuronal cell membrane (Fig. 4C).

### KEGG pathway analysis of F0 rats cadmium exposure on mRNAs in hippocampal tissue of F1 rats

**Fig. 5:**
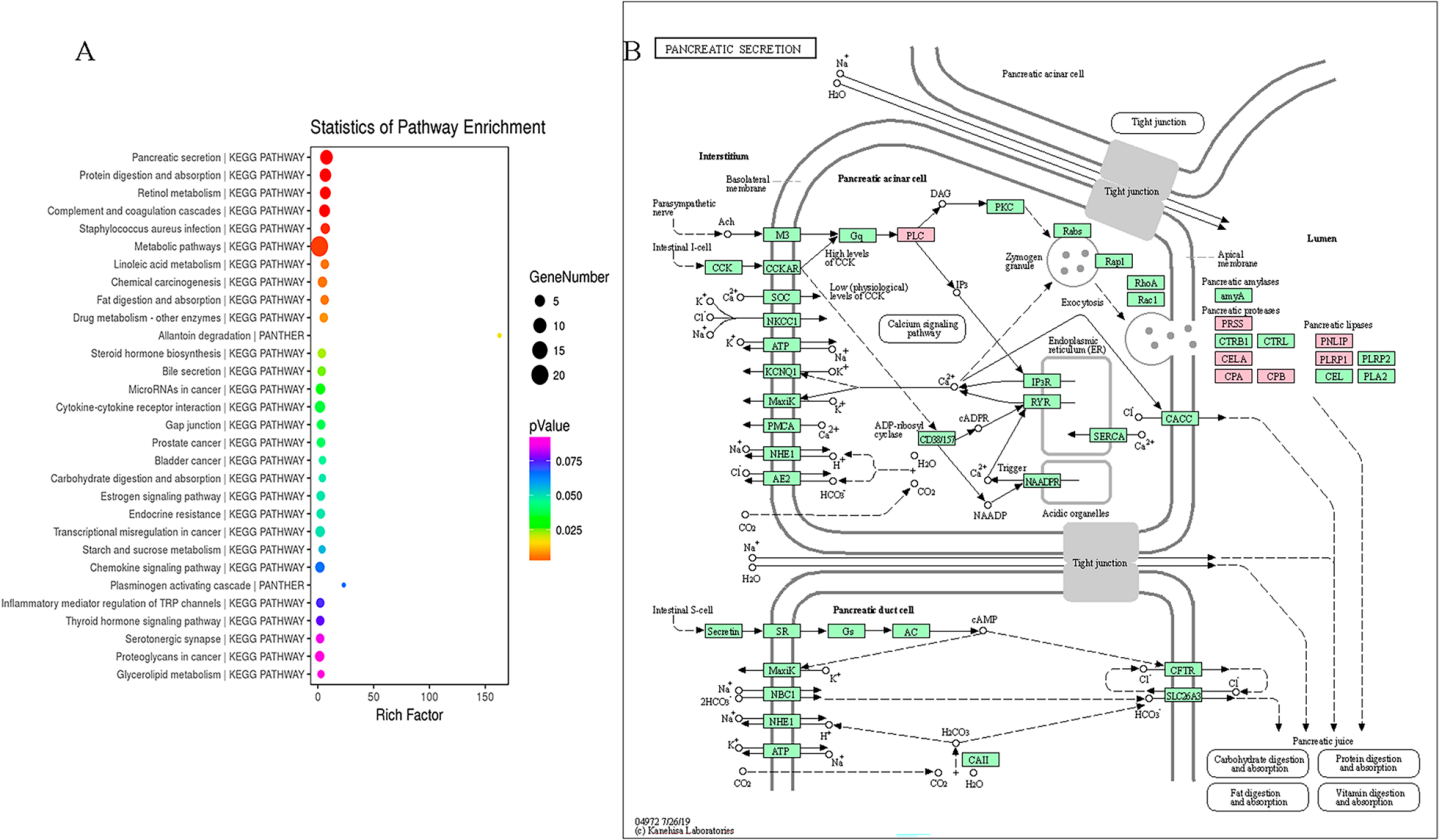
KEGG analysis of differentially expressed mRNA. **A** Pathway enrichment analysis bubble plot. The bubble diagram drawn according to the p value, the size of the bubble indicated the number of genes annotated to this functional entry by the differential gene, and the color corresponds to the p value in the enrichment analysis result. **B** Pancreatic secretory pathway. Differentially expressed genes in the pathway are marked in pink

Pathway enrichment analysis of differentially expressed mRNAs was performed by KEGG pathway analysis, and bubble plots listed the top 30 significantly enriched pathways (Fig. 5A). Using *P* ≤ 0.05 as the standard, 8 pathways enriched by differentially expressed mRNA were found. The results showed that the effects of maternal cadmium exposure on offspring rats might be involved in metabolic pathways, “pancreatic secretion”, “protein digestion and absorption”, “retinol metabolism”, “complement and blood coagulation cascades”, and “Cytokine-cytokine receptors”. The most relevant pathway is “pancreatic secretion”. So we show pancreatic secretory pathway of KEGG and marked differentially expressed genes in this map, as Fig. 5B.

### The effect of maternal cadmium exposure on the differential expression of mRNA in the hippocampus of F1 rats may involve the pancreatic secretion pathway.

**Fig. 6:**
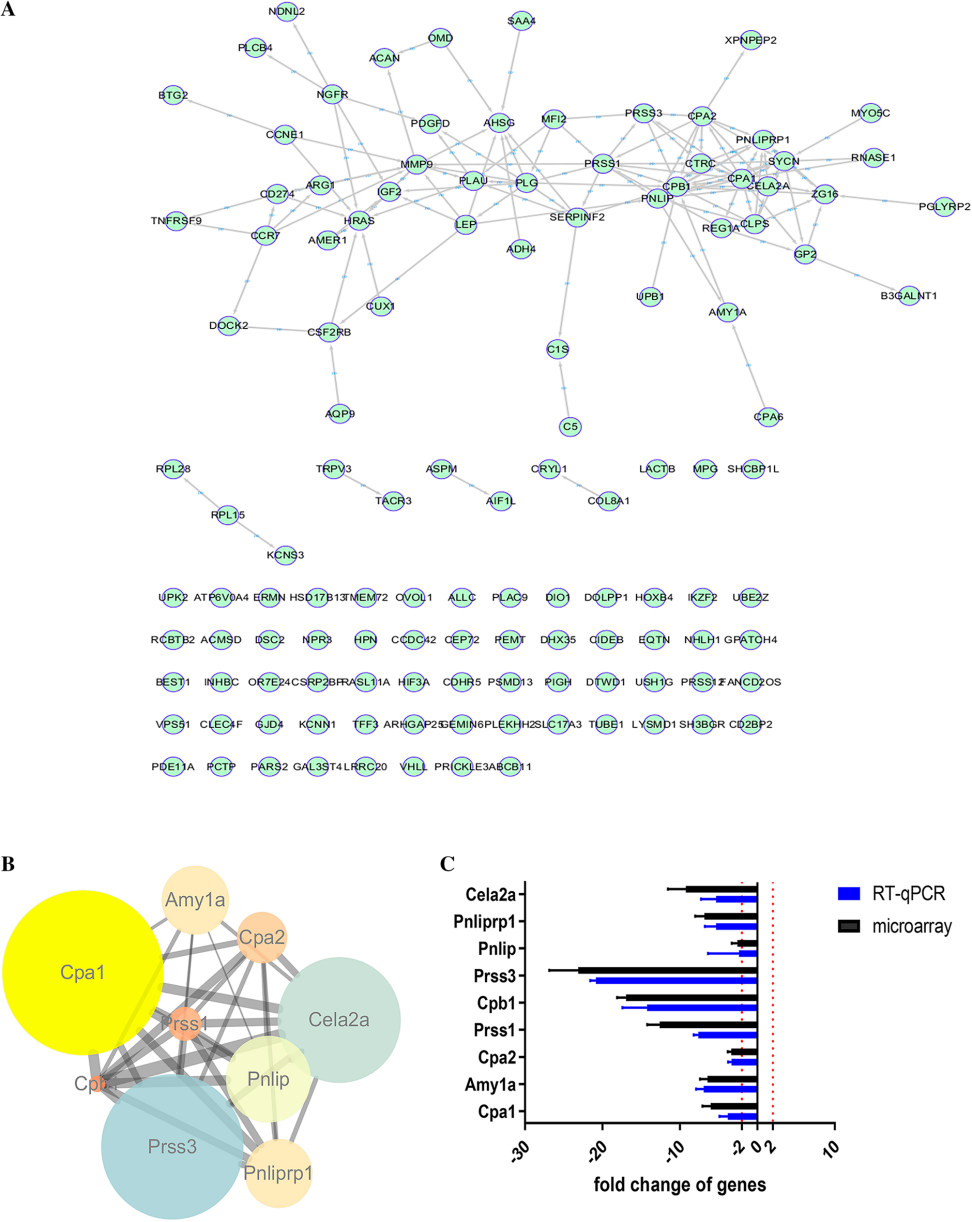
The protein–protein interaction (PPI) network and the gene-mRNA network. **A** PPI map of differentially expressed mRNA genes; **B** map of differentially expressed mRNA enriched in pancreatic secretion-related genes; **C** RT-qPCR and Microarray

The differentially expressed mRNA in the hippocampal tissue of F1 rats caused by maternal cadmium exposure was imported into the STRING APP plug-in of Cytoscape software, and a differentially expressed protein–protein association network was constructed (Fig. 6A). Based on the previous KEGG analysis, the pathway that exhibits the highest number of differential genes is the 'pancreatic secretion' pathway, as depicted in Fig. 5A. The correlation of cadmium exposure to the differentially expressed mRNA enriched in the "pancreatic secretion pathway" in the hippocampal tissue of F1 rats was listed in STRING (Fig. 6B). Validation of the transcription levels of lncRNAs and mRNAs to confirm the expression of lncRNAs and mRNAs, and RT-qPCR analysis was applied to verify the RNA-seq results in pancreatic secretory pathway. These results, revealing that the RNA-seq results were consistent with the RT-qPCR results, verified that the RNA-seq results were reliable (Fig. 6C).

### Analysis of the hub genes in F1 rats hippocampus by F0 rats exposure to Cadmium

**Fig. 7:**
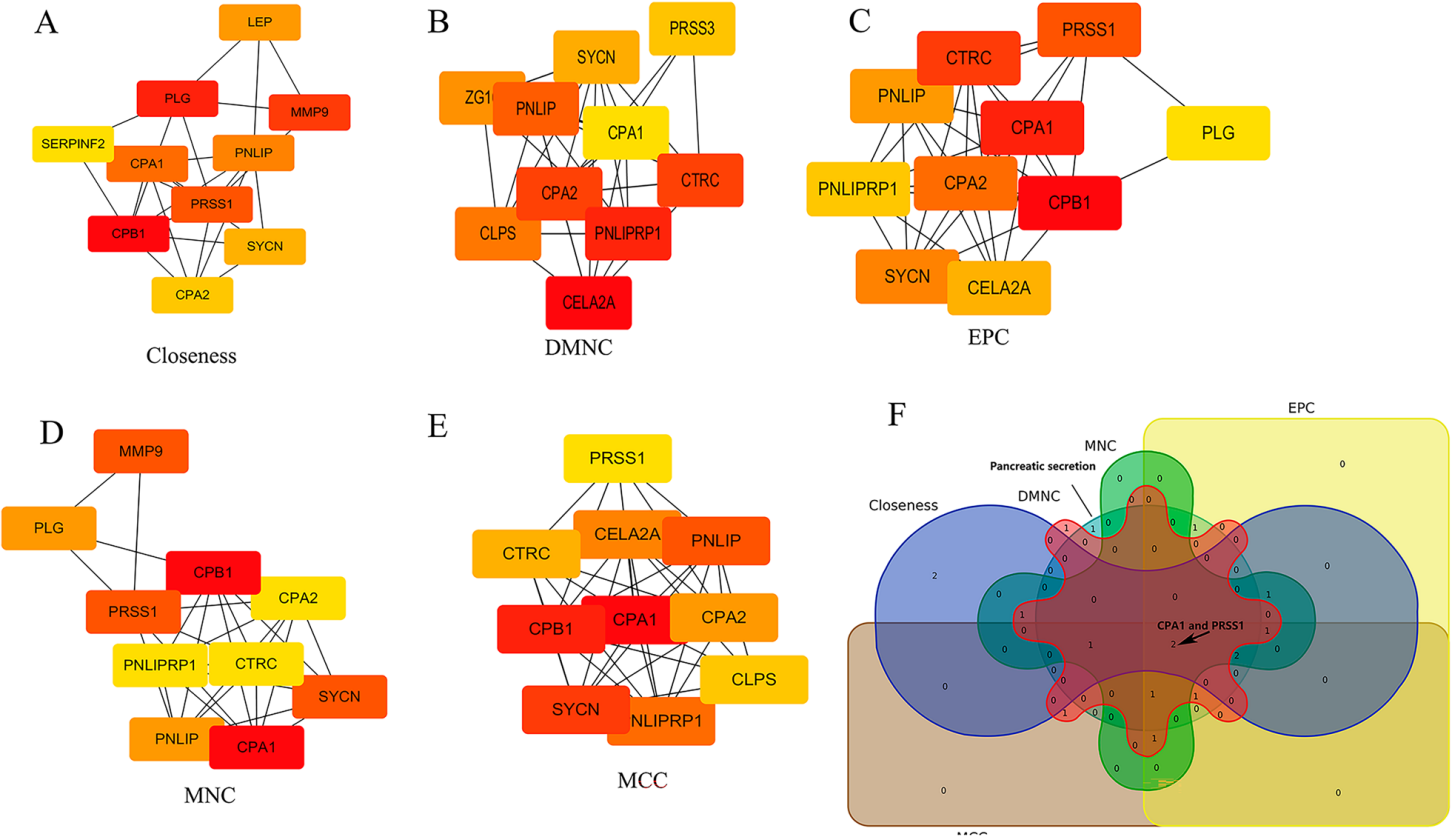
The protein–protein interaction (PPI) network and screening hub target gene. **A** Closeness; **B** DMNC; **C** EPC; **D** MNC; **E** MCC; **F** Venn diagram

The top 10 genes among the differentially expressed genes were calculated by using the five topological analysis algorithms of Closeness, DMNC, EPC, MNC and MCC of the cytoHubba, and the interaction relationship of the top ten genes was drawn by Cytoscape as shown in Fig. 7A–E). The venn diagrams were used to identify important hub genes, and combined with key genes enriched in the "pancreatic secretion pathway", various algorithms and screening results suggested that Prss1 and Cpa1 were important Hub gene, as shown in Fig. 7F. Therefore, it is suggested that the two genes play an important role in the mechanism of cadmium-induced cognitive impairment in F1 rats.

### Construction of lncRNA-mRNA weighted co-expression network

**Fig. 8:**
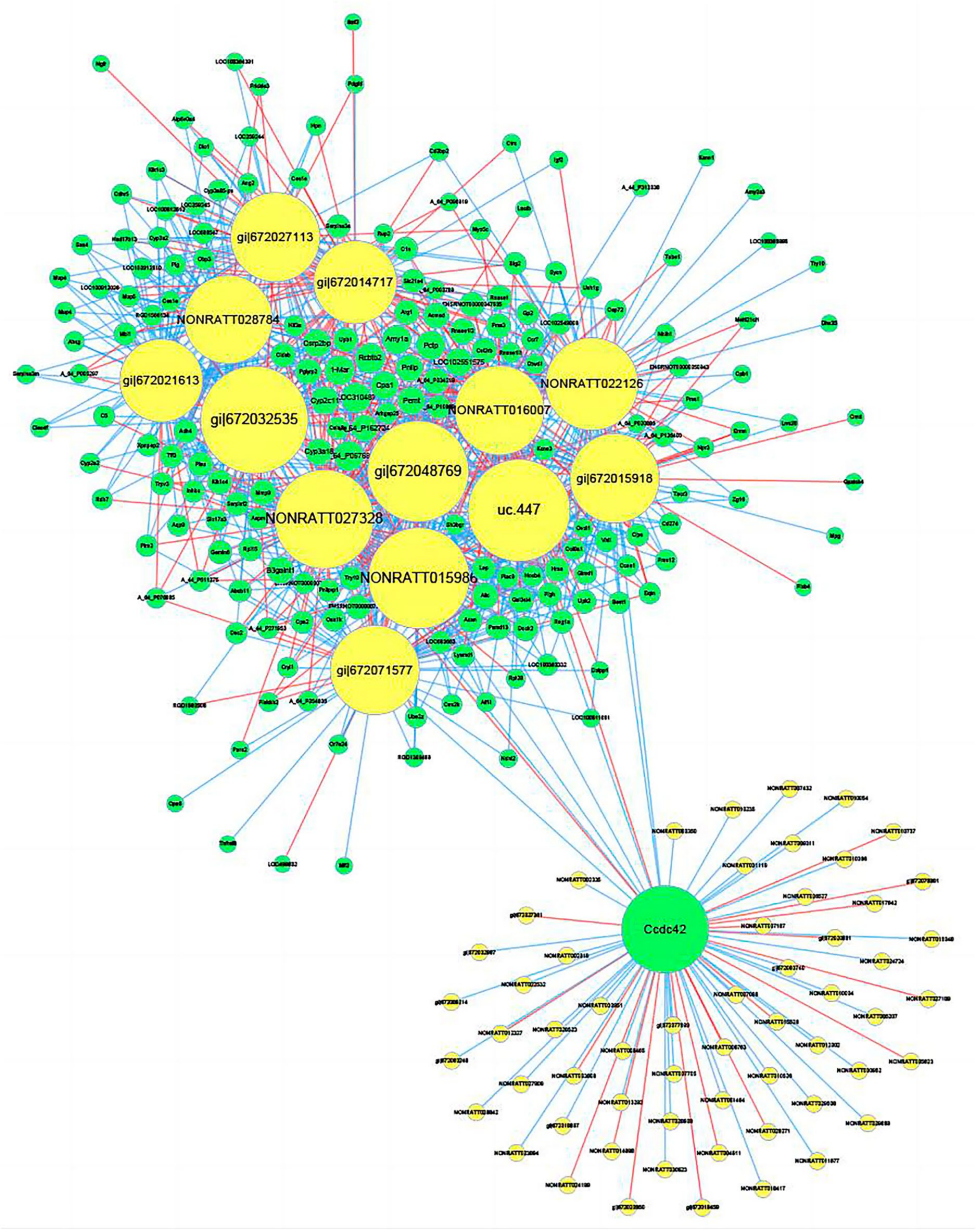
LncRNA-mRNA co-expression network. Yellow circles represent lncRNAs, green circles represent mRNAs, the size of the circles represents the degree of genes in the network (number of neighbors), red lines represent positive correlations, and blue lines represent negative correlations

In this study, we first constructed the co-expression network of coding and non-coding genes between the cadmium treatment group and the control group, in which lncRNAs and mRNAs were differentially expressed, and selected lncRNAs and mRNAs with Pearson correlation ≥ 0.99, and then used cytoscape software to construct a co-expression network. To express the network, construct the top 1000 relationship pairs with the largest correlation in each of the above groups, and the results are shown in Fig. 8.

### Transcription factor prediction

**Fig. 9:**
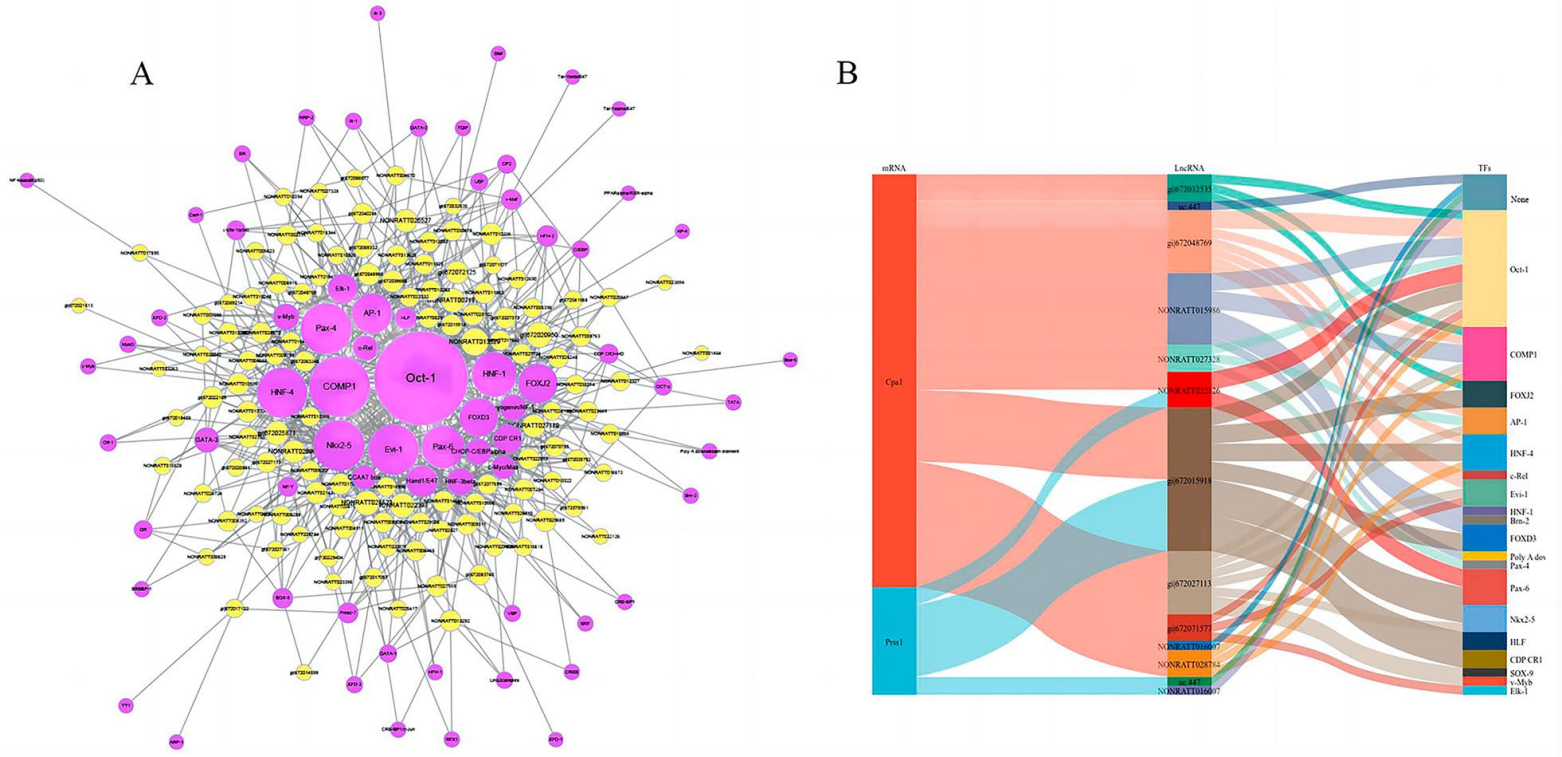
Transcription factor prediction. **A** Transcription factor prediction network. Purple circles represent transcription factors, yellow circles represent LncRNAs, the size of the circles represents the degree of genes in the network (number of neighbors), and straight lines represent correlations. **B** Sankey diagram

Transcription factors (TFs) are the key regulators of the transcriptional expression of multiple genes in various biological processes. A total of about 1893 lncRNA-transcription factor (TF) pairs were found, corresponding to 1449 TFs. Then, the most credible top 1000 LncRNA-TF pairs (the lowest p value and FDR) generated a core network, as shown in Fig. 9A, and it was found that most of these potential trans-regulated lncRNAs were mainly involved in pathways regulated by five transcription factors: c-Myc/Max, Oct-1, HNF-4, GR, v-Myb, and the sankey diagram as Fig. 9B, the gene list as Additional file 2.

## Discussion

The effects of first-generation cadmium exposure on the cognitive abilities of the second generation have been a subject of controversy [18]. Our animal experiments demonstrated that exposure to Cd during non-pregnancy could impair the cognitive abilities of offspring rats. In this study, we constructed a model of cadmium poisoning in first-generation rats through gavage. In the water maze experiment, the offspring of Cd-treated rats showed increased levels of panic and anxiety, as well as a faster swimming speed upon entering the water, compared to the offspring of control rats. The escape latency of offspring exposed to Cd for 6 consecutive days was higher than that of the offspring of control rats. In the spatial probe experiment, the spatial memory of the offspring of Cd-treated rats was significantly decreased compared to the offspring of control rats. The results of the water maze experiment suggest that first-generation Cd exposure impairs the cognitive and memory abilities of its offspring. These findings are consistent with previous studies that have shown maternal cadmium exposure to have an impact on neurodevelopment in male offspring [19].

A consistent trend supporting this conclusion has been observed in previous population studies. For example, a study in Bangladesh involving 1,305 mother–child pairs found that higher maternal urinary Cd levels during pregnancy were associated with reduced size, performance, and verbal IQ in children at the age of 5 years [20]. Similarly, a Chinese study found a negative association between prenatal Cd exposure (measured by Cd concentrations in cord blood) and the full-scale intelligence quotient (IQ) at 4.5 years of age [21]. However, most previous studies have focused on the effects of Cd exposure on cognitive performance in the second generation, specifically examining the impact of maternal Cd exposure during pregnancy on offspring. There have been fewer studies investigating the effects of Cd exposure in the first generation (including males) on cognitive disability. Nevertheless, Cd exposure in the first generation is not influenced by gender and may be attributed to occupational factors, with men being more likely to be exposed to cadmium contamination. In the context of pregnancy, the exposure of women to Cd is expected to be reduced due to changes in lifestyle behaviors such as reduced alcohol consumption, dietary modifications, and decreased occupational exposure. Therefore, when studying the developmental toxicity of the next generation, particularly its impact on cognitive abilities and learning, it is important to prioritize the assessment of Cd exposure before pregnancy. This study emphasizes the significance of including both males and females in the analysis.

The most important pathway in KEGG pathway enrichment analysis was “pancreatic secretion”. Therefore, maternal Cd exposure may affect islet secretion-related pathways in the hippocampus of offspring, consistent with previous studies reporting that islet cells may be related to cognitive ability [22]. Neuroprotective effects of pancreatic islet transplantation into the central nervous system, as well as neurotrophic and angiogenic factors, with potential neuroprotective properties [23]. Using various algorithms, we identified Cpa1 and Prss1 as hub genes. First generation cadmium exposure may reduce the gene and protein expression levels of Cpa1 and Prss1 in the hippocampus of offspring rats. Therefore, we speculated that Cpa1 and Prss1 were most likely to be the key genes involved in hippocampal damage in the offspring of Cd-treated rats. But the relationships between the expression levels of Cpa1, Prss1 and cognitive ability were unclear. However, the roles of Cpa1 and Prss1 in the pancreatic function are clear. All of them are key genes in the "Pancreatic Secretion" pathway. As previously mentioned, pancreatic secretion is associated with cognitive ability. Therefore, this study suggested that Cd exposure during non-pregnancy may impair the cognitive ability of offspring; the mechanism underlying the effects of Cd on cognitive ability may be related to pancreatic secretion-related pathways, especially those related to Cpa1 and Prss1. However, further experiments are needed to clarify the functional role of Cpa1 and Prss1 in the cognitive ability of the offspring of Cd-exposed rats.

To investigate the potential involvement of LncRNAs in the cognitive effects of cadmium on rat offspring, we constructed a co-expression network consisting of the top 1000 pairs of LncRNAs and mRNAs. This network aimed to elucidate the biological functions and regulatory mechanisms of LncRNAs [24]. Within the co-expression network, we observed that Cpa1 was co-expressed with 11 LncRNAs (gi|672032535, uc.447, gi|672048769, NONRATT015986, NONRATT027328, NONRATT022126, gi|672015918, gi|672,027,113, gi|672071577, NONRATT016007, NONRATT028784). In addition, we found a correlation between Prss1 and 4 relatively unexplored LncRNAs (uc.447, NONRATT022126, gi|672015918, NONRATT016007). These findings provide valuable insights for future studies investigating the impact of LncRNAs on cognitive function in offspring exposed to cadmium. Furthermore, we constructed a Sankey diagram to illustrate the relationship between Cpa1, Prss1, and the associated LncRNAs and transcription factors.

The study presents some preliminary results regarding the potential effects of cadmium on cognitive impairment in offspring. However, further functional validation is required to validate these conclusions. In addition, the molecular pathways identified by some differential genes may be biased and more pathways may be enriched. Despite these limitations, the results of this study are significant for two main reasons. Firstly, the effects of cadmium exposure on offspring cognition were explored through animal experiments. Secondly, it provides valuable insights for future studies on the mechanism of cadmium-induced cognitive impairment in offspring. Overall, this study supports further research into cognitive impairment and the damaging effects of cadmium.

### Supplementary Information

**Additional file 1: Figure S1.** Establishment of the cadmium poisoning rat model and gene chip sample data. (A) The cadmium accumulation in the hippocampus among F0 and the F1 rats (Cd)-treated and control rats. Data were normalized to total protein content.Principal component analysis (PCA) plot of differentially expressed (B) LncRNA and (C) mRNA in hippocampal tissue.

**Additional file 2.** Gene list of this Sankey diagram.

## Data Availability

All data generated or analyzed during this study were included in this published article.

## Abbreviations

Cd: Cadmium
qRT-PCR: Quantitative real-time polymerase chain reaction
KEGG: Kyoto Encyclopedia of Genes and Genomes
GO: Gene Ontology
MCC: Maximal Clique Centrality
TFs: Transcription factor
PCA: Principal component analysis
PPI: Protein–protein interaction
